# Indications and use of cone beam computed tomography in children and young individuals in a university-based dental hospital

**DOI:** 10.1186/s12903-023-03784-4

**Published:** 2023-12-21

**Authors:** Rovshan Ismayılov, Beste Özgür

**Affiliations:** Department of Pediatric Dentistry, Hacettepe University Faculty of Dentistry, Altındağ, Ankara, 06100 Turkey

**Keywords:** Cone beam computed tomography, Pediatric dentistry, Indications, Dental radiology

## Abstract

**Background:**

The aim of this study was to evaluate the indications of cone beam computed tomography (CBCT) in children and young individuals in a university-based dental hospital and their association with age, gender and field of view.

**Methods:**

7131 CBCT scans, taken during 3-year period, were reviewed and a total of 649 pediatric patients (0–18 years) with complete request forms were included. Data related to gender, age, referring department, CBCT indications, field of view (FOV), region of interest (ROI), need for re-exposure and patients received more than one CBCT examination were recorded.

**Results:**

The mean age was 13.57 ± 3.52 years and “malocclusion and dentofacial anomaly” (28.7%) was the most common clinical indication. Facial trauma, dental trauma and supernumerary tooth in males; “malocclusion and dentofacial anomaly” and implant planning in females were recorded more frequently compared to other gender. Maxilla was the most frequently monitored ROI (35.1%) for patients. Small (≤ 10 cm) FOV was preferred in 58.1% of all patients. Large FOV was selected in the majority of patients who underwent CBCT scan for “malocclusion and dentofacial anomaly” (89.6%). The repeated scans constituted 2.3% of patients and 105 patients (16.2%) underwent multiple CBCT scans on different dates for mainly orthodontic follow-up.

**Conclusions:**

The justification of CBCT scans was not fully compatible with current guidelines and mainly larger FOV was preferred. The number of CBCT examination in children and young individuals tends to increase.

**Trial registration number:**

Not applicable.

## Background

The cone beam computed tomography (CBCT) started a new era in 3D (axial, sagittal, coronal) maxillofacial imaging by providing high-quality images that can be obtained with a relatively compact device and lower cost compared to the conventional computed tomography (CT) [1–3]. Cross-sectional image sets obtained in different planes with a single scan, and the multiplanar reconstruction ability resulted in more accurate diagnosis which has increased the popularity of CBCT in all fields of dentistry [4–6]. However, gradual replacement of 2D panoramic imaging by CBCT without a valid justification is a growing concern, particularly considering the 2–45 times higher radiation dose, which is not negligible [7]. The international organization bodies and scientific groups on radiation safety emphasize that the stochastic effects of low-energy ionizing radiation are a permanent potential risk to induce cancer by causing DNA damage and mutation [4, 8].

Justification of radiographic examination in children is crucial as they are 2–10 times more susceptible to ionizing radiation compared to adults [9–11]. There is a greater need for judicious use in dentistry, as dentists perform radiographic examination more often on children unlike most medical professions [11]. Recently, the European Academy of Paediatric Dentistry (EAPD) published a policy document for prescribing dental radiographs in children and adolescents, which suggested to limit radiation exposure according to the ALADAIP principle (As Low As Diagnostically Achievable being Indication-oriented and Patient-specific) [12]. Also, repeated CBCT examinations should be avoided due to higher risk of stochastic effect in pediatric population [9, 10].

The European evidence-based guideline (SEDENTEXCT) [11] specified recommendations for the usage of CBCT but the current level of evidence regarding pediatric indications still remains limited [13]. EAPD [12] reported that CBCT may be indicated in case of severe dentoalveolar trauma, root resorption, cleft lip and palate patients, developmental disorders (amelogenesis imperfecta, etc.), cysts and benign tumors, dental anomaly (dens invaginatus, dilaceration, etc.), autotransplantation, unerupted, impacted and ectopic teeth. Whilst, some clinicians support routine use of CBCT in orthodontic treatment [14], large volume CBCT was not recommended by the SEDENTEXCT guidelines [11]. CBCT can also be used to assess dento-alveolar side-effects of orthodontic treatment in children, including external root resorption [15]. Furthermore, CBCT should not be the first-line imaging method in children and adolescents [12]. There is only one study evaluating the knowledge of pediatric dentists regarding CBCT and it concluded that about one third of the participants had no knowledge [16]. Mostly nonexistent or low-grade evidence addressing the indications of CBCT in young populations is discouraging, as dental radiography is frequently utilized in pediatric dental practice [4, 12]. The aim of this research was to investigate the indications of CBCT in children and young individuals, who underwent a CBCT scan in a university-based hospital. Additionally, the distribution of the CBCT indications according to different categories (age, gender, field of view, region of interest) was evaluated.

## Methods

The study protocol of this retrospective and cross-sectional study was approved by the Institutional Review Board of Hacettepe University (Protocol No: GO 20/665). The study was conducted following Strengthening the Reporting of Observational studies in Epidemiology (STROBE) guidelines.

Power analysis was performed using G*power. In the light of a similar study [17], the minimum sample size was reached 285 patients with 0.05 α error, 0.85 effect size and 0.95 power (1 − β) (one-tailed hypothesis).

**Fig. 1:**
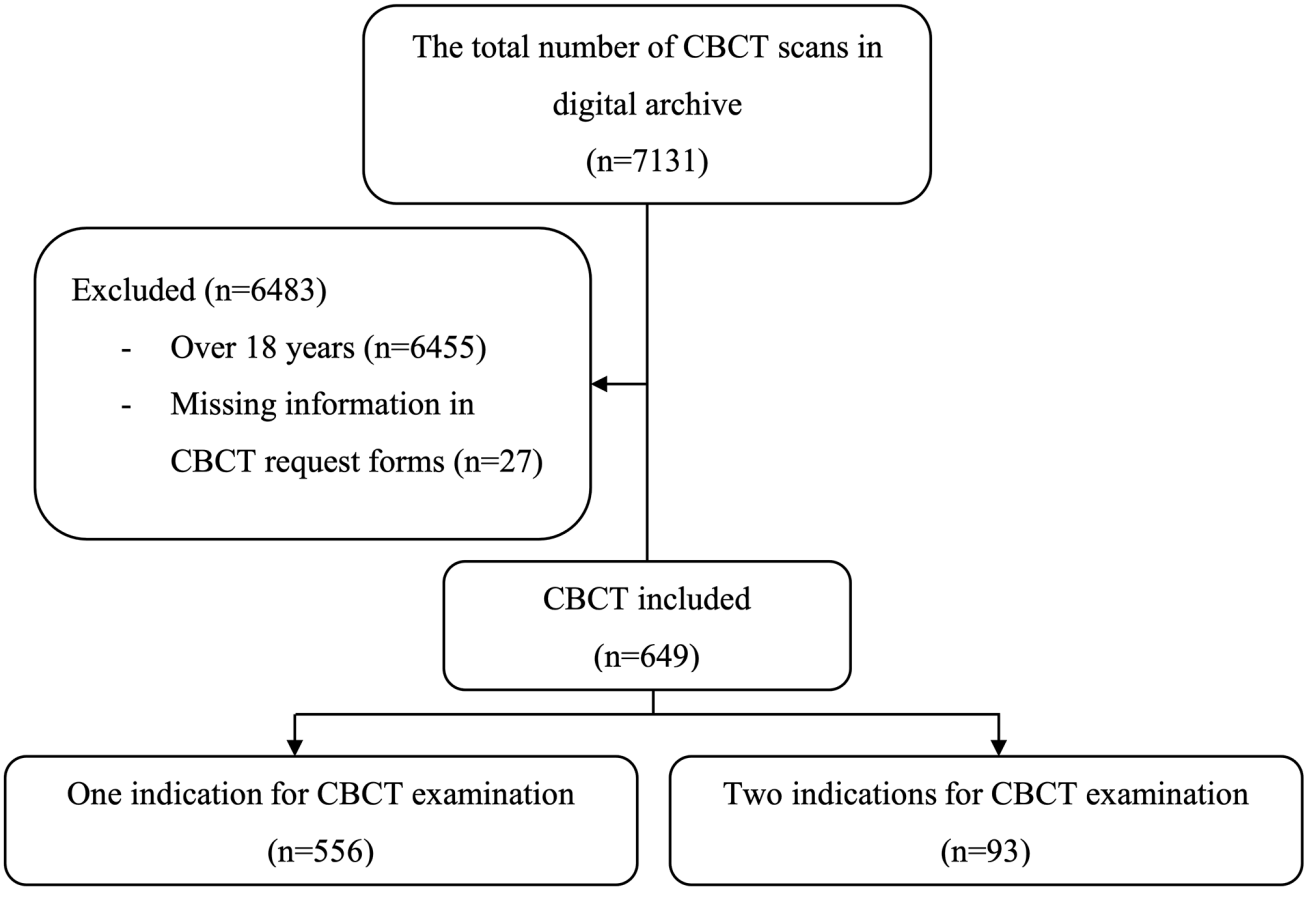
The flow of participants

The digital oral radiology archive of Hacettepe University Faculty of Dentistry was the source of data studied herein (Nucleus MBS, Monad, Istanbul, Turkey). Between January 2018 and January 2021, the CBCT request forms of patients, who were 18 years old and younger, were analyzed and those with missing information were excluded (Fig. 1). A researcher (RI) recorded the following data into Microsoft Excel® (Microsoft Inc., WA, USA): gender, age at the time of examination (years), CBCT indication, the department, which requested the CBCT examination, the reason for the repeated scans on the same day and the patients who received more than one CBCT examination. Patients were categorized into three age groups according to the dentition stage as follows; 0–6 years (primary dentition), 7–12 years (mixed dentition) and 13–18 years (permanent dentition) [18].

The images of all patients were obtained with i-CAT Next Generation CBCT Unit (Imaging Sciences International, Hatfield, PA, USA) with the use of “scout view”. The CBCT images with complete request forms, were evaluated by BO and RI using a 5 K retina display, 27-inch monitor (iMac, Apple Inc., CA, USA) for confirming the stated indication and also recording the technical parameters. One of these parameters, the field of view (FOV), is a cylindrical volume that determines the shape and size of the reconstructed image. The CBCT images were grouped based on size of FOV as small (height: ≤10 cm, diameter: 16 cm), medium (height: 11–15 cm, diameter: 16 cm) and large (height: 23 cm, diameter 17 cm). The other parameter, region of interest (ROI), was categorized as maxilla, mandible, maxilla + mandible and craniofacial.

Statistical analysis was performed using SPSS 23.0 software (IBM Corp., Chicago, IL). Categorical variables were shown as numbers and percentages. The effects of gender, age and FOV on CBCT indications were evaluated with Pearson’s chi-square and Fisher’s exact tests. The level of significance was set at 5%.

## Results

**Fig. 2:**
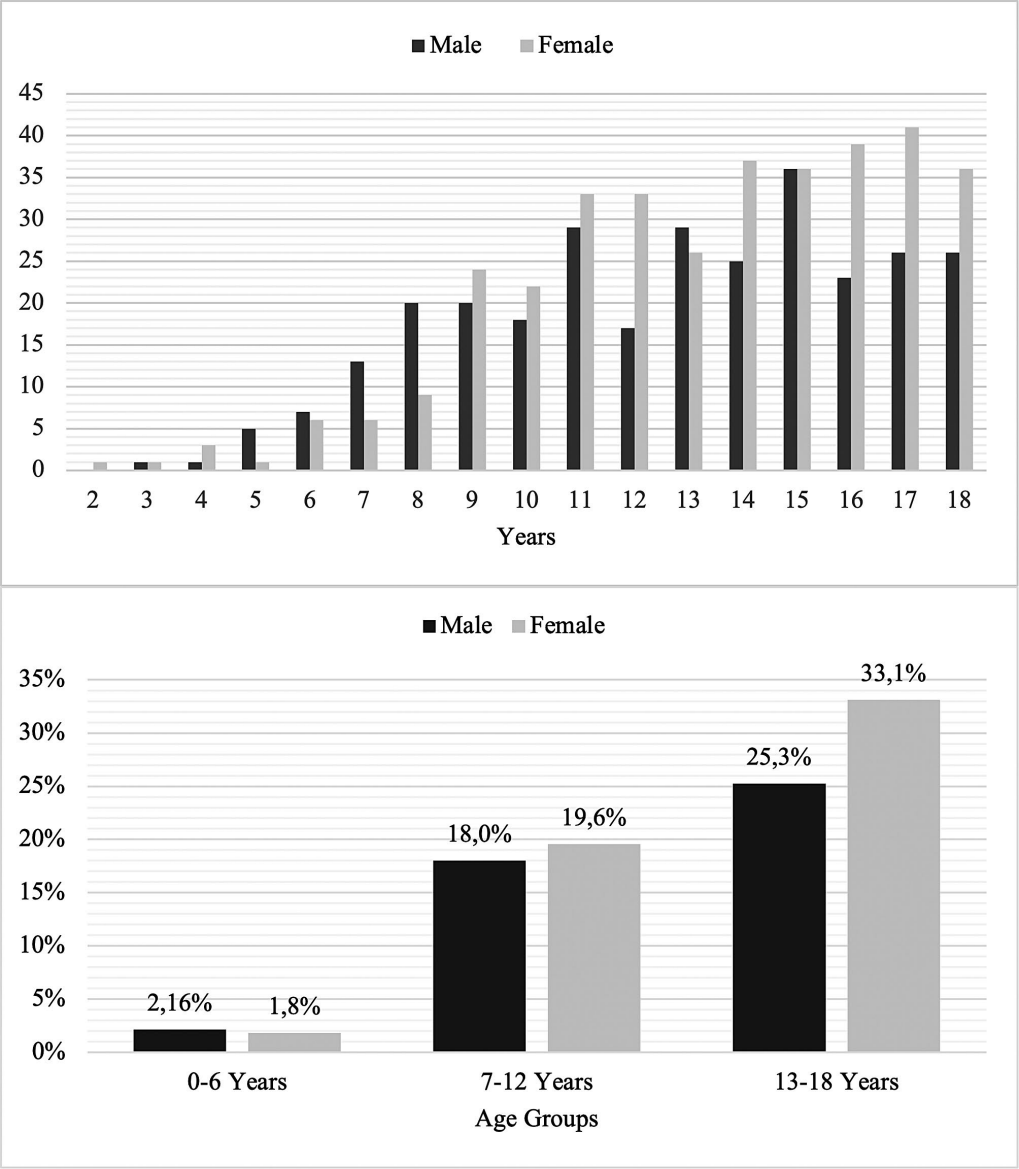
Distribution of patients by age (or age groups) and gender

During 3-year study period, a total of 676 CBCT scans were requested for patients aged ≤ 18 years, which represented 9.5% of all CBCT examinations. A total of 649 patients were included, 354 (54.5%) females and 295 (45.5%) males, and the mean age was 13.57 ± 3.52 years (between 2.83 and 18.67 years) (Fig. 2). The youngest patient was 2.8 years old and, underwent CBCT scan for temporomandibular joint (TMJ) and partial mandibular hypoplasia (malocclusion and dentofacial anomaly).

The majority of patients (n = 379, 58.4%) were in the 13–18 years age group, which had the most apparent difference in gender distribution (females: 33.1%, males: 25.5%) (Fig. 2). Although CBCT examinations in 2019 increased compared to 2018 for mixed and permanent dentition groups, a pronounced decrease was observed in 2020 due to shutdowns caused by COVID-19 pandemic (Table 1). Fourteen types of CBCT indications were specified in the referrals (Table 2). The “malocclusion and dentofacial anomaly” (28.7%) was the most frequent clinical indication, followed by cysts and other bone pathology (20.1%) and localization of impacted tooth (16.9%). In 93 patients two different reasons were stated for the imaging request. The reviewed CBCT images were compatible with the reasons of request stated by the dentists. The “malocclusion and dentofacial anomaly” and implant planning in females; facial trauma, dental trauma and supernumerary tooth in males were significantly higher than other gender (*p* < 0.05, Table 2).

**Table 1:** Annual trends of CBCT scans by different age groups

	Year							
Age group	2018		2019		2020		Total	
	n	%	n	%	n	%	n	%
0–6 years	11	5.4	9	2.8	6	4.7	26	4.0
7–12 years	75	37.1	120	37.6	49	38.3	244	37.6
13–18 years	116	57.4	190	59.6	73	57.0	379	58.4
**Total**	202^a^	100	319^b^	100	128^c^	100	649	100

a-b: %57.9 increase, b-c: %59.9 decrease, a-c: %36.6 decrease

**Table 2:** Association between CBCT indications and gender

CBCT indications	Total		Female		Male		
	n	%	n	%	n	%	*p*
Malocclusion and dentofacial anomaly	192	28.7	121	63.0	71	37.0	0.005^*^
Dental anomaly	13	1.9	6	46.2	7	53.8	0.539
Localization of impacted tooth	113	16.9	68	60.2	45	39.8	0.186
Cysts and other bone pathology	134	20.1	64	47.8	70	52.2	0.077
Facial trauma	18	2.7	3	16.7	15	83.3	0.001^*^
Dental trauma	18	2.7	5	27.8	13	72.2	0.021^*^
Cleft lip and palate	21	3.1	10	47.6	11	52.4	0.517
Temporomandibular joint pathology	20	3.0	15	75.0	5	25.0	0.062
Localization of foreign object	1	0.1	1	100	0	0	-
Supernumerary tooth	76	11.4	32	42.1	44	57.9	0.020^*^
Root resorption	12	1.8	4	33.3	8	66.7	0.136
Implant planning	19	2.8	16	84.2	3	15.8	0.008^*^
Endodontic assessment	28	4.2	16	57.1	12	42.9	0.778
Periodontal assessment	3	0.4	1	33.3	2	66.7	-
**Total (N = 668 indications)	668	100	362	54.2	306	45.8	

Pearson’s chi-square test, *Statistically significant = *p* < 0.05, ** Multiple indications may be selected in a patient

“Malocclusion and dentofacial anomaly”, facial trauma, implant planning and TMJ pathology were more common indications in 13–18 years age group; whereas dental anomaly and supernumerary tooth were recorded more frequently in 7–12 years age group. The association between these CBCT indications and age groups was statistically significant (Table 3). For all CBCT indications, the lowest number of CBCT examination were present in primary dentition group (0–6 years). In this group, the most commonly identified reason for CBCT request was cyst and other bone pathology (n = 9, 32.1%). Small FOV (height ≤ 10 cm) was selected in the majority of patients (58.1%), followed by large (30.5%) and medium (11.4%) FOVs. The association between CBCT indications and size of FOV (small, medium, large) was evaluated. This was highly significant for “malocclusion and dentofacial anomaly” (89.6% large FOV), localization of impacted tooth (75.2%, small FOV), cysts and other bone pathology (75.4%, small FOV), facial trauma (94.4%, medium FOV), dental trauma (61.1%, medium FOV), temporomandibular joint pathology (80.0%, medium FOV), supernumerary tooth (84.2%, small FOV) and endodontic assessment (89.3%, small FOV) (*p* < 0.001) (Table 3). Also, there was a statistically significant association between implant planning and the size of FOV, indicating that mostly small FOV (84.2%) was preferred (*p* < 0.05) (Table 3).

**Table 3:** Association between CBCT indications and age group or field of view size

CBCT indications	Primary dentition		Mixed dentition		Permanent dentition			Small FOV		Medium FOV		Large FOV		
	0–6 years		7–12 years		13–18 years			≤ 10 cm		11–15 cm		23 × 17 cm		
	n	%	n	%	n	%	*p*	n	%	n	%	n	%	*p*
Malocclusion and dentofacial anomaly	3	1.6	63	32.8	126	65.6	^1^0.017*	15	7.8	5	2.6	172	89.6	^1^0.000*
Dental anomaly	0	0	10	76.9	3	23.1	^2^0.023*	12	92.3	1	7.7	0	0	-
Localization of impacted tooth	1	0.9	40	35.4	72	63.7	^1^0.125	85	75.2	13	11.5	15	13.3	^1^0.000*
Cysts and other bone pathology	9	6.7	55	41.0	70	52.3	^1^0.094	101	75.4	33	24.6	0	0	^1^0.000*
Facial trauma	3	16.7	4	22.2	11	61.1	^1^0.014*	0	0	17	94.4	1	5.6	^1^0.000*
Dental trauma	2	11.1	6	33.3	10	55.6	^1^0.294	7	38.9	11	61.1	0	0	^1^0.000*
Cleft lip and palate	1	4.8	7	33.3	13	61.9	^1^0.913	13	61.9	2	9.5	6	28.6	^1^0.652
Temporomandibular joint pathology	3	15.0	5	25.0	12	60.0	^1^0.029*	3	15.0	16	80.0	1	5.0	^1^0.000*
Localization of foreign object	0	0	0	0	1	100	-	1	100	0	0	0	0	-
Supernumerary tooth	6	7.9	45	59.2	25	32.9	^1^0.000*	64	84.2	9	11.9	3	3.9	^1^0.000*
Root resorption	0	0	7	58.3	5	41.7	^2^0.331	12	100	0	0	0	0	-
Implant planning	0	0	0	0	19	100	^1^0.001*	16	84.2	3	15.8	0	0	^1^0.009*
Endodontic assessment	0	0	8	28.6	20	71.4	^1^0.265	25	89.3	3	10.7	0	0	^1^0.000*
Periodontal assessment	0	0	1	33.3	2	66.7	-	2	66.7	1	33.3	0	0	-
**Total (N = 668 indications)	28	4.2	251	37.6	389	58.2		356	53.3	114	17.1	198	29.6	

^1^Pearson’s chi-square test, ^2^Fisher’s exact test, *Statistically significant = *p* < 0.05, ** Multiple indications may be selected in a patient

**Fig. 3:**
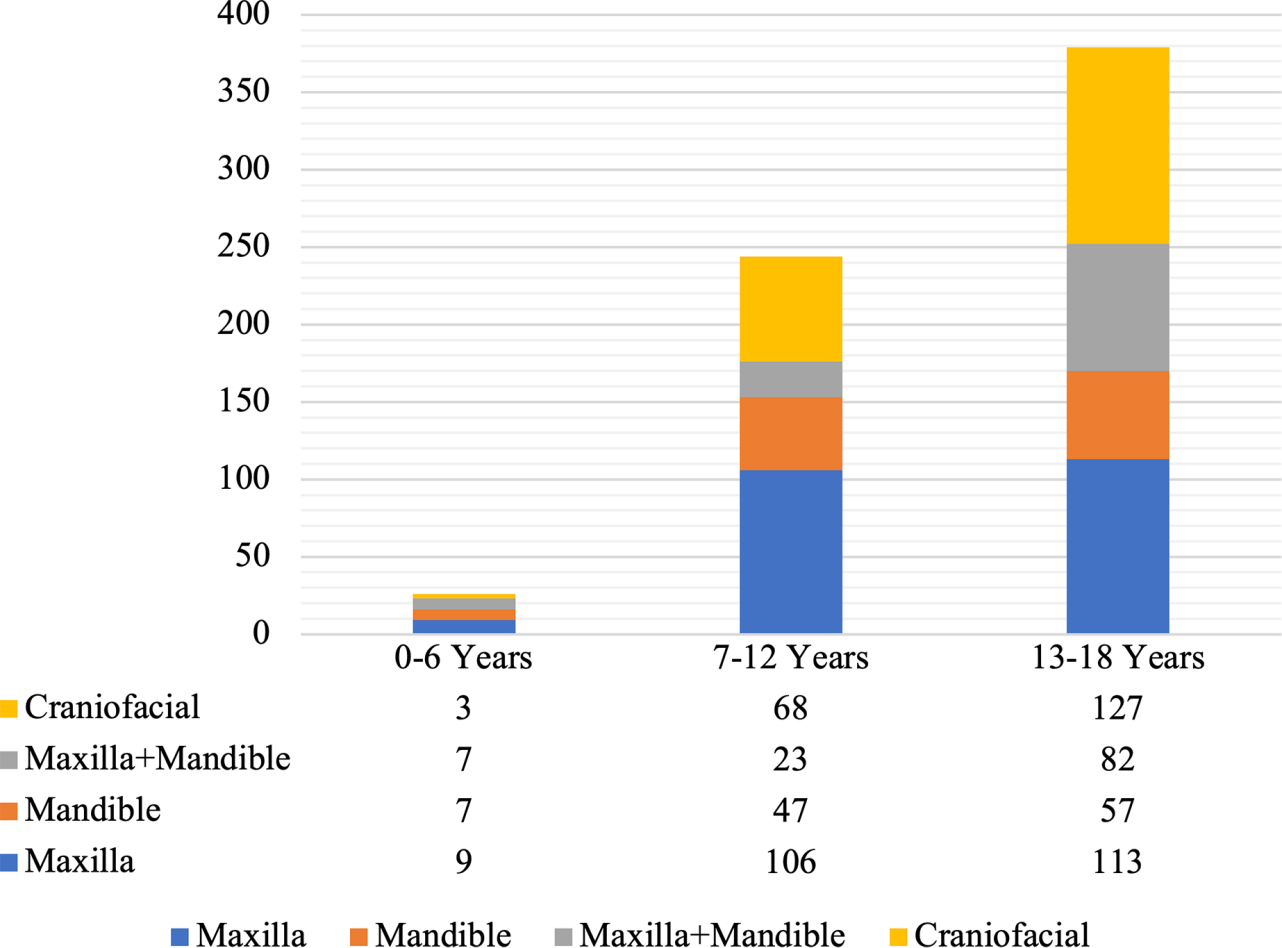
Distribution of region of interest per age groups

Maxilla (35.1%) was the most frequently imaged region in patients, followed by the craniofacial region (30.5%). The distribution of ROI according to the age groups revealed that the craniofacial region (33.5%) was the most commonly monitored region in 13–18 years age group, while in 0–6 (34.6%) and 7–12 (43.4%) years age groups, maxilla was most commonly targeted for imaging (Fig. 3). Maxilla was also the most common ROI for localization of foreign object and root resorption (Table 4). In terms of other ROIs; the mandible for periodontal assessment, maxilla and mandible for temporomandibular joint pathology and the craniofacial region for dentofacial anomalies were recorded with a higher incidence (Table 4).

**Table 4:** Distribution of region of interests (ROI) in terms of CBCT indications

CBCT indications	Maxilla			Mandible		Maxilla + Mandible		Craniofacial	
	n	%	n		%	n	%	n	%
Malocclusion and dentofacial anomaly	15	7.8	1		0.5	4	2.1	172	89.6
Dental anomaly	10	76.9	2		15.4	1	7.7	0	0
Localization of impacted tooth	55	48.7	21		18.6	22	19.4	15	13.3
Cysts and other bone pathology	35	26.1	67		50.0	32	23.9	0	0
Facial trauma	1	5.6	2		11.1	14	77.7	1	5.6
Dental trauma	6	33.3	2		11.1	10	55.6	0	0
Cleft lip and palate	15	71.4	0		0	0	0	6	28.6
Temporomandibular joint pathology	0	0	1		5.0	18	90.0	1	5.0
Localization of foreign object	1	100	0		0	0	0	0	0
Supernumerary tooth	49	64.5	9		11.9	15	19.7	3	3.9
Root resorption	11	91.7	1		8.3	0	0	0	0
Implant planning	14	73.7	2		10.5	3	15.8	0	0
Endodontic assessment	22	78.6	4		14.3	2	7.1	0	0
Periodontal assessment	0	0	2		66.7	1	33.3	0	0
**Total (N = 668 indications)	234	35.0	114		17.1	122	18.3	198	29.6

**Fig. 4:**
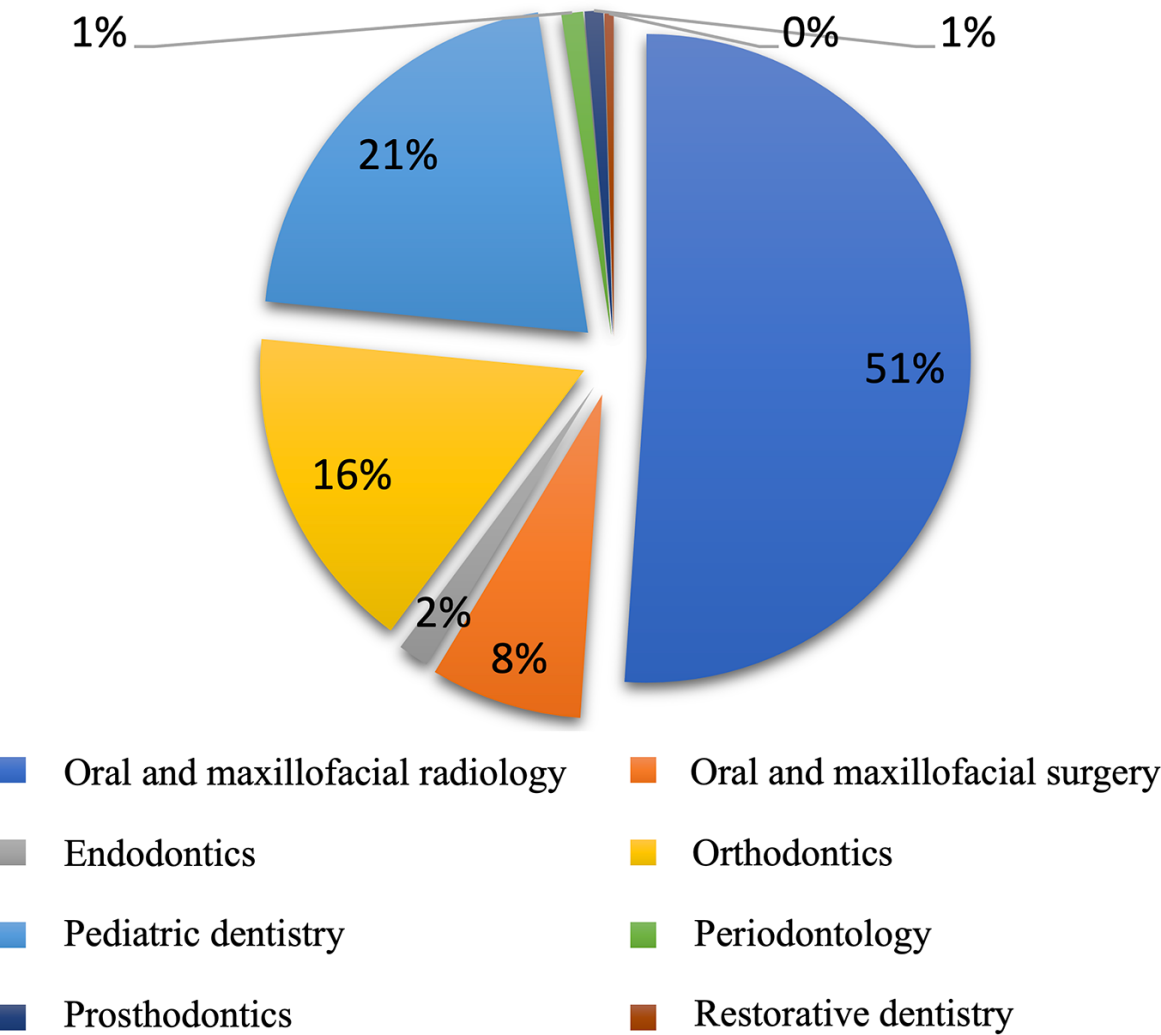
Distribution of departments that requested the CBCT examination

Figure 4 shows the distribution of CBCT requests made by different departments. The highest number of CBCT request was made by oral and maxillofacial radiology (n = 331), followed by pediatric dentistry (n = 136) and orthodontics (n = 107). The majority of patients, who were referred for CBCT from oral and maxillofacial radiology and orthodontics were in 13–18 years age group, while most of the referrals from pediatric dentistry were in 7–12 years age group. The association between these departments and age groups was statistically significant (*p* < 0.05).

Repeated scans on the same day constituted 2.3% of all patients (n = 3 in 0–6 years, n = 7 in 7–12 years, n = 5 in 13–18 years age group) and motion artefact was the only identified reason. The correlation between age groups and motion artefact could not be evaluated due to insufficient number of individuals. 105 patients (16.2%) underwent multiple CBCT scans on different dates for orthodontic follow-up (51.4%), surgical follow-up (42.0%) and other reasons (6.6%). Among these patients, one CBCT examination was required in 76.4% following the initial scan, while two or more additional scans were obtained in 23.6%. The shortest time period between consecutive scans belonged to a patient, who had complicated facial trauma and received a follow-up scan one day after surgery. A patient (16 years 8 months) experienced seven CBCT examinations within 23 months, which was the highest number of repeated scans, and the initial CBCT indication was cyst and other bone pathology. The minimum and maximum time elapsed between two consecutive follow-up scans was one and 1646 days, respectively (mean: 494 days).

## Discussion

The technological advancement, increasing functionality and the cost-effective accessibility of CBCT have made its use widespread in contemporary dentistry. Following CBCT evaluation, some studies reported revision in clinical decision, initially determined by conventional imaging [19–21]. On the other hand, CBCT does not always provide high diagnostic accuracy or a clear benefit for the patient [22–24]. However higher reimbursement compared to intraoral imaging in some countries and easy administering may have led to a frequent use of this technique [25]. Since the literature regarding CBCT referrals in pediatric dentistry is limited [5, 17, 25, 26], the radiation protection guidelines indicate the need for an update in the light of new studies [11, 13, 27].

The central location of the Hacettepe University Faculty of Dentistry which shares the same campus with Hacettepe University Hospitals, may be the reason for the diversity in terms of profile and the number of the patients. Gallichian et al. [18] showed that the number of CBCT scans for children aged 16 or under increased each year, with an 160% total increase rate between 2015 and 2019. In another study conducted on patients younger than 19 years, the number of CBCT scans did not increase annually but the total increase rate between 2017 and 2020 was 223.8% [28]. In the present study, an increase of 57.9% was found between 2018 and 2019, while the number of CBCT scans decreased by 36.6% in 2020 compared to 2018. The strict COVID-19 lockdown in Turkey started on March 11, 2020 and the majority of dentists were assigned to the filiation (chain of transmission) unit, which reduced the number of appointments during the slow release. On the other hand, in similar retrospective studies (including 2 to 6 years period) conducted on children and young individuals, the number of CBCT scans ranged from 79 to 449 [5, 17, 18, 28–30]. Despite the lockdown, 3-year retrospective data of this study resulted in high number of CBCT examinations (n = 676), which can be attributed to the aforementioned characteristics of the hospital, where the present study was conducted.

In the present study, children and young adults constituted a smaller proportion (9.5%) of all CBCT examinations and the mean age of patients was 13.57 ± 3.52 years. Looking at the age range of patients, the mean age was between 11.00 and 13.7 years in similar literature [5, 17, 18, 25, 28–30]. The results of the Yiğit at al. (mean age 14.32 years, range 6–18 years) [28], Hidalgo Rivas et al. (mean age 13.1 years, range 5–17 years) [5] and İşman et al. (mean age 13.42 years, range 2–17 years) [17] were correlated with the present study in terms of mean age, but the pediatric age groups of these studies constituted a higher proportion (13.65–38.7%) of all CBCT scans. İşman et al. [17] reported that the high prevalence of the inbreeding coefficient in the southeast region of Turkey could have resulted with dentofacial anomalies, and consequently higher proportion of pediatric age group (38.7%). In addition, non-justified use of CBCT was also identified in their study [17]. In a study by Gümrü et al. [30], 5.1% of all patients was ≤ 14 years old (mean age 11.15 years, range 3–14 years). The inclusion of patients up to 18 years old in our study may explain the higher number of children and young individuals.

The classification of various “justification for referral” in similar studies makes it difficult to compare the results. Localization of impacted teeth (along with possible root resorption of the adjacent teeth) [5, 17, 19, 25, 28–30] has stood out as the most common CBCT indication for children and young adults. Bone pathology (cysts and other lesions) [5, 17, 19, 30], dental anomalies (supernumerary teeth, atypical tooth morphology, etc.) [19, 29–31], surgical assessment [18], “malocclusion and dentofacial anomalies” [17], and localized applications of CBCT for the developing dentition [18, 29] were among the most frequent CBCT indications. These results were consistent with the present study. Gümrü et al. [30] reported that the main indication for the use of CBCT, similar to present study, was “malocclusion and dentofacial anomaly” (38.5%), which contradicts with the majority of the literature [5, 18, 25, 28, 29]. In DIMITRA and SEDENTEXCT guidelines, craniofacial CBCT is not recommended as a standard method for orthodontic-related radiological assessment [11, 13]. For complex cases of skeletal abnormality, particularly those requiring combined orthodontic/surgical management, large volume CBCT may be justified in planning the definitive procedure [11]. Since the present study was conducted in a tertiary referral hospital with high patient admissions, higher number of patients presenting with the need of advanced treatment can be expected. Another possible reason may be frequent request of craniofacial CBCT scans in orthodontics, which is not compatible with the current guidelines. Bone pathosis (9.6%) [28]; syndromes (0.4%), trauma (0.9%) [30]; localization of a foreign object (0.3%), airway evaluation (0.6%), TMJ problems (1.5%) [17]; post-operative complications following dental extraction (0.2%), determining presence or absence of the teeth (0.2%) [25]; dental trauma (0.6%), TMJ (1.0%) [5]; periodontics (1.0%) and TMJ (1.0%) [29] have been among the least recorded CBCT indications in previous studies. Although CBCT has been widely regarded as a method to visualize the upper airway with less radiation [32], it was not a CBCT indication the present study.

There were more females (54.5%) in the present study, which was similar to the results by Hidalgo-Rivas et al. (53.1%) [5] and Yiğit et al. (50.9%) [28]; and in contrast to the results by Van Acker et al. (44.3%) [29], İşman et al. (46.4%) [17], Hajem et al. (45.7%) [25], Gümrü et al. (41.2%) [30]. The association between gender and CBCT indication was rarely evaluated in pediatric population and no significant correlation was found [17, 30]. The “malocclusion and dentofacial anomaly” was the most common CBCT indication in this study, and significantly higher numbers of this indication in females may be attributed to higher prevalence of malocclusion in this gender [33, 34]. The literature, which reports higher frequency for facial trauma [35, 36], dental trauma [37] and supernumerary teeth [38, 39] in males, is in line with the findings of the present study. Approximately, the growth of the facial skeleton is completed at 16–17 years in females and 21–22 years in males [40, 41] and usually considered as the sublimit for implant planning and placement [42]. The exclusion of patients above 18 years of age in our study explains the significantly lower prevalence of implant planning in males (15.8%) compared to females.

Considering the effect of dentition stage on CBCT indication, the number of CBCT scans requested in the permanent dentition period was higher (58.4%) and this finding was consistent with similar studies [5, 17, 28, 29]. The referral reason (malocclusion and dentofacial anomaly) of the youngest patient was different to that reported as trauma by Gümrü et al. (3-year-old) [30] and İşman et al. (2-year-old) [17]. In the only similar study, which evaluated the correlation between the identical age groups as ours and CBCT indication, “malocclusion and dentofacial anomaly” was significantly more frequent in permanent dentition than those in the other dentition stages; while trauma and localization of impacted teeth were significantly higher in mixed and permanent dentition [17]. Van Acker et al. [29] found a significant difference between age groups (< 10 years and 10–12 years old) regarding the indication of “developing dentition-localized”, which consisted of the greatest part out of orthodontic referrals. For the age group younger than 10 years old, the odds for that indication was about 29 times lower compared to the odds for the 10–12 years old age group, which typically involves second transitional period and the permanent dentition [29]. The studies, concluding that the need for fixed orthodontic treatment/orthognathic surgery [43] and jaw fractures due to facial trauma [36, 44] increased during permanent dentition stage (13–18 years old age group), support the results of the present study. Implant planning was only present in the permanent dentition group (100%), as implants usually are not placed before facial growth is complete [42, 45]. Another significantly higher CBCT indication in the permanent dentition group was TMJ problem (60%). Although CBCT is known to be a reliable method for the assessment of osseous defects of the TMJ [46], magnetic resonance imaging (MR) is the method of choice, where monitoring of the TMJ disc is required [11]. Clinical symptoms and degenerative changes due to TMJ dysfunction in young patients increase with age, which complies with our result [47, 48]. During mixed dentition stage, it is possible to diagnose various dental anomalies and eruption disturbances that may occur related to supernumerary teeth [49, 50], and this could be the reason of significant increase of dental anomaly and supernumerary tooth related indications.

In similar studies, most of the patients were referred from department of oral and maxillofacial radiology (53.6%) [28], pediatric dentistry (36.3%) [30] or general practice and specialized caregivers (43%) [25]. In the present study, referral of some patients from various departments to the department of oral and maxillofacial radiology with manual CBCT request form and performing of the digital data entry in this department may have resulted in more CBCT requests (51.0%).

Similar to the literature (2.5–6.5%), only 2.3% of the patients had repeated scans. İşman et al. [17] found a negative association between age and motion artefacts, which may reflect poor cooperation, anxiety, or long scanning time in younger children. In a systematic review, a consensus on the fact that children and adolescents often move during the CBCT examination was reached [51]. On the other hand, it is also known that the image quality is related to accumulated number, duration and complexity of movements [52]. Another reason for repeated scans, is inadequate FOV size [5, 17]. The scout view obtained before CBCT scans in the present study, prevented inadequate coverage of the area and resulted in no repeated scans for this reason [25]. The most commonly reported reasons for follow-up examinations were similar to literature in children and young individuals (orthodontic follow-up, orofacial clefts and syndromes) [17, 30], although more patients (16.2%) had multiple CBCT scans in this study. Gümrü et al. [30] reported that most of the patients (82.7%) with multiple scans underwent one follow-up examination, which was in line with the findings of the present study (76.4%).

Variances in study methodologies regarding the classifications of ROI and FOV, complicate comparison of the findings. The region of interest (ROI) has been grouped to include sextants separately or in different combinations (four, five or six) [5, 17, 18, 28, 30]. The most frequently referred ROI was anterior maxilla [5, 18, 28, 30]. The frequent referrals of patients with impacted tooth, cleft lip palate, and supernumerary tooth, which are usually localized in maxilla [36, 39], may explain this finding. In similar studies, more than one sextant was examined in 11% of the individuals [18] and extended ROI (more than two contiguous sextants or more than one non-contiguous sextant) was examined in 18.5% [5]. The frequent use of extended ROI (maxilla + mandible and craniofacial) in our study contradicted with these results. Gallichan et al. [18] evaluated the distribution of ROI according to age groups (≤ 6 years, 7–12 years, 13–16 years) and reported that the most commonly requested sextant was upper anterior in all age groups. The distribution of ROI according to CBCT indications was evaluated and the results were similar with other studies for bone pathology [28, 30], dental anomaly [28, 30], and impacted tooth [30]; while different ROIs were reported for endodontic assessment (anterior mandible) [28], trauma (regional) [30], orofacial clefts (craniofacial) [30], and impacted tooth (posterior mandible) [28].

CBCT equipment should offer a choice of volume sizes and clinicians must use the smallest that is compatible [11]. Hidalgo Rivas et al. [5] reported that the largest FOV available for i-CAT (23 × 17 cm) was never used and for 3D Accuitomo device a small FOV, mainly the smallest available (4 × 4 cm), was used in 88.8% of the patients. These results concurred with Van Acker et al. (FOV: 5 × 5.5 cm, 81%) [29]. Hajem et al. [25] concluded that smallest FOV (4 × 4 cm) was preferred in 48% of the scans, while large FOV (17 × 17 cm) was used in only one patient among 617. The i-CAT Next Generation, used in the present study, has limited ability to alter FOV diameter but is versatile in altering height. While the largest FOV was selected in 198 cases (30.5%), the fact that the smallest FOV (8 × 8 cm) was not used contradicted with similar studies. In terms of the largest FOV usage, lower (20.5%) [30] or higher (74.2%) [17] percentages were reported by other researchers from Turkey. İşman et al. [17] stated that in 70% of CBCT scans, unnecessarily high FOV was used. In our study, the correlation between “malocclusion and dentofacial anomaly” and large FOV was highly significant. This practice can be deemed controversial with regards to SEDEXTCT guideline, which recommends very critical consideration, particularly in pediatric age group [11]. Particularly outside of Europe, large volume CBCT was reported to be used as a routine tool for orthodontic-related radiological assessment [53, 54]. In some countries, CBCT regulations state that a licensed specialist in oral and maxillofacial radiology must confirm that CBCT scan is justified and then supervise the examination [55]. The authors of this study believe that such practice may not only limit unnecessary administration but also help determine the appropriate FOV size for the given indication.

## Conclusion

To the best of the authors’ knowledge, this study included the largest cohort of CBCT examination in pediatric patients. The most common indication was “malocclusion and dentofacial anomaly”. Significant associations between CBCT indication and gender, age group or field of view size were found. The justification of CBCT scans was not fully compatible with current guidelines and mainly larger FOV was preferred. Meticulous consideration by clinicians is required to avoid unnecessary radiation exposure, particularly in children and young individuals.

## Data Availability

The datasets generated during and/or analysed during the current study are available from the corresponding author on reasonable request.

## List of abbreviations

ALADAIP: As low as diagnostically achievable being indication-oriented and patient-specific
CBCT: Cone beam computed tomography
CT: Computed tomography
EAPD: European academy of paediatric dentistry
FOV: Field of view
ROI: Region of interest
TMJ: Temporomandibular joint
